# Unravelling the grey zone: submaximal-to-peak stress echocardiography enhances diagnostic accuracy in differentiating early dilated cardiomyopathy from physiological adaptation

**DOI:** 10.1186/s44156-026-00123-5

**Published:** 2026-06-15

**Authors:** Sarandeep K. Marwaha, Joyee Basu, Raghav Bhatia, Hamish Maclachlan, Saad Fyyaz, Maria Teresa Tome Esteban, Elijah R. Behr, Gherardo Finocchiaro, Aneil Malhotra, Sanjay Prasad, Sabiha Gati, Michael Papadakis, Sanjay Sharma

**Affiliations:** 1 Cardiovascular Research Institute, City St George’s, University of London, Cranmer Terrace, London, SW17 UK; 2 St George’s University Hospitals NHS Foundation Trust, London, UK; 3 Manchester Metropolitan University, Manchester, UK; 4 Royal Brompton and Harefield NHS Foundation Trust, London, UK

**Keywords:** Athletes, Dilated cardiomyopathy, Grey zone, Left ventricular ejection fraction, Stress echocardiogram

## Abstract

**Background:**

Participation in regular endurance exercise may be associated with physiological left ventricular (LV) dilatation and concomitant low resting LV ejection fraction (LVEF), a phenotype indistinguishable from early dilated cardiomyopathy (DCM) on resting imaging alone and termed the “grey zone”. Stress echocardiography has emerged as a potential arbiter and has been proposed to resolve this dilemma.

**Objectives:**

We evaluated the diagnostic accuracy of stress echocardiography in distinguishing physiological from pathological LV dilatation and assessed whether incorporating submaximal-to-peak contractile reserve improved discriminatory value in athletes in the grey zone.

**Methods:**

Of the 182 athletic individuals, 62 control athletes with an enlarged LV and normal LVEF, 58 athletic DCM individuals, and 62 grey zone athletes underwent stress echocardiography using a semi-supine bicycle. In addition to the ability to augment LVEF ≥ 10% from rest to maximal exercise, we evaluated the ability to augment LVEF from submaximal exercise (80% of maximal heart rate) to peak exercise.

**Results:**

Resting LV dimensions did not differ significantly amongst the groups. Control athletes had higher resting LVEF than grey zone athletes and DCM individuals (62.1% vs 52.1% and 53.1%; *p*=<0.001). Control and grey zone athletes showed greater ΔLVEF from rest to peak exercise than DCM individuals (21% and 19.2% vs 4.9%; *p* < 0.001). Most control (98.3%) and grey-zone athletes (90.3%) achieved a ΔLVEF ≥ 10% from rest to peak exercise compared with 20.6% of athletic DCM individuals. Control and grey zone athletes also revealed a mean increase in LVEF from submaximal to peak exercise of 7.5% and 3.8%, respectively, whereas DCM individuals showed a mean LVEF decline of −4.3% (*p* < 0.001). Although 20.6% of DCM individuals demonstrated a ΔLVEF ≥ 10% from rest to peak, only a single DCM individual augmented LVEF from submaximal to peak exercise. Failure to increase LVEF ≥ 10% from rest to peak exercise identified DCM with 79.4% sensitivity and 98.3% specificity. Combining this inability with a failure to augment LVEF from submaximal to peak exercise improved the sensitivity to 98.2% and specificity to 98.4%.

**Conclusion:**

Reliance on rest-to-peak augmentation of ≥10% alone risks misclassification. Introducing submaximal-to-peak augmentation enhances diagnostic precision by identifying virtually all individuals with DCM while preserving specificity.

## Introduction

Endurance athletes, particularly males engaging in long-distance cycling, running, rowing, swimming, and canoeing, frequently exhibit enlarged cardiac dimensions which significantly exceed the predicted upper limits observed in the general population and overlap with those detected in patients with dilated cardiomyopathy (DCM) [1]. A low resting LV ejection fraction (LVEF) of 45–54% is also recognised in a proportion of athletes with an enlarged LV diameter [2, 3], raising the possibility of DCM. An erroneous diagnosis of DCM in an ostensibly healthy athlete may result in advice to refrain from intensive exercise. In contrast, a false diagnosis of an athlete’s heart in an individual destined to develop progressive DCM is a missed opportunity to initiate prognostically proven therapies to minimise the risk of sudden cardiac death, perform regular surveillance and risk stratification to evaluate disease progression and prescribe safe exercise [4].

We previously demonstrated that exercise echocardiography reveals abnormal findings in almost 80% of athletic DCM individuals. Specifically, most athletic DCM individuals could not increase their LV ejection fraction > 11% from rest to peak exercise. Our index was derived from a relatively small study focussing solely on male athletes and demonstrated a high specificity. However, limitations remained, given the serious consequences of an incorrect diagnosis, 23% of athletic DCM individuals also fulfilled this criterion, highlighting the potential for diagnostic error in this population [5]. The European Society of Cardiology (ESC) Sports Cardiology guidelines currently recommend an increase in LVEF of ≥10% from rest to peak exercise as an accepted marker of good myocardial reserve. This parameter is often applied clinically to help differentiate physiological LV enlargement from DCM in athletes [6].

In this study, we sought to examine the role of stress echocardiography in a larger cohort of athletic DCM individuals and ostensibly healthy athletes with an enlarged LV and a depressed resting LV ejection fraction (“grey zone athletes”). Apart from evaluating our previously established parameter for good myocardial reserve (Δ LVEF > 11% from rest to peak exercise and the ESC recommended threshold of ≥10%) [5, 6], we assessed the additional role of the ability to augment LV ejection fraction between the height of submaximal exercise (80% of the predicted maximal heart rate) and peak exercise.

Ethical approval was obtained by the Health Research Authority, sponsored by St George’s Hospital Research Ethics Committee, in August 2021. All procedures complied with the Declaration of Helsinki. Written informed consent was obtained from all participants prior to enrolment, and all participant information sheets and consent forms were approved by the REC.

## Methods

### Subjects

All participants in the study were recruited from the inherited cardiac diseases and sports cardiology units at St George’s University Hospital, London (UK) and the Brompton Hospital, London (UK) between August 2021 and July 2023. Inclusion criteria were asymptomatic status and persistent participation in vigorous exercise for >4 hours per week as determined by a health questionnaire and consultation. Control and DCM individuals were matched for age and sex. Individuals with comorbidities potentially impacting cardiac size and function, such as hypertension and valvular heart disease, and those who were unable to perform a supine or upright exercise test on a bicycle, were excluded.

### Control athletes

Eighty-one potential control athletes (dilated LV with a normal LV ejection fraction ≥ 55%) were approached from various national cardiac screening programmes linked with the sports cardiology unit at St George’s University Hospital, including football, rugby, triathlon and cycling. Of these, 62 agreed to participate.

### Athletic dilated cardiomyopathy individuals

582 DCM individuals were reviewed and identified from the inherited cardiac disease clinic databases at St George’s University and the Brompton Hospitals. Dilated cardiomyopathy was defined as the presence of LV dilatation and systolic dysfunction unexplained solely by coronary artery disease, hypertension or valvular heart disease. Sixty-four individuals had an LVEF of 45–54% and participated in regular recreational or competitive sports. Of these, 58 agreed to participate in the study, including 17 individuals with restoration of normal LV function after pharmacological treatment. Thirty-one (53%) had idiopathic DCM, with previously documented moderate to severe LV impairment at diagnosis, 23 (42.6%) had familial DCM, 2 (3.7%) individuals had previous myocarditis, 1 (1.8%) had postpartum cardiomyopathy, and 1 (1.8%) had recovered from anthracycline-related cardiomyopathy. Of the 21 (40%) individuals tested genetically, 8 (15.3%) had a diagnostic pathogenic variant for DCM.

### Grey-zone athletes

Sixty-eight consecutive athletes falling within the grey zone (dilated LV with an LV ejection fraction of 45–54%), often considered an indeterminate group, were prospectively recruited from the sports cardiology unit at St George’s, University of London, which receives national referrals for athletes with potential cardiac pathology. Grey zone athletes were considered in the absence of symptoms, pathological arrhythmias, history of progressive LV dysfunction, or secondary causes (coronary artery disease, hypertension, valvular disease). Of these, eight athletes with mild LV fibrosis on CMR were also included, given its occurrence in some apparently healthy endurance athletes [7].

### Definitions

An enlarged LV cavity was defined using the British Society of Echocardiography guideline recommendations (LV diameter ≥ 57 mm in males and ≥52 mm in females or indexed volume ≥ 80 ml/m^2^ in males and ≥71 ml/m^2^ in females) [8]. Borderline/low left ventricular systolic function was defined as a resting LVEF between 45 and 54%.

### Investigations

All subjects were evaluated using a health questionnaire, 12-lead ECG, stress echocardiogram, cardiopulmonary exercise stress test, 24-hour Holter monitor, and

cardiovascular magnetic resonance (CMR) scan. The methodology for ECG, CPET, 24-hour Holter and CMR is discussed elsewhere [5]. CMR was used to assess for major fibrosis (subendocardial, mid-wall, or subepicardial involvement). ECGs were interpreted using the international guidelines for athletes [9]. Anterior T-wave inversion was defined as T-wave inversion extending beyond lead V2, i.e V3 and V4, in the absence of an incomplete right bundle branch block pattern. Inferior T-wave inversion was defined as T-wave inversion in two or more inferior leads (II, III, and aVF). An isolated premature ventricular complex on the resting ECG was not classified as abnormal.

### Stress echocardiography

Participants were asked to discontinue inhibitory chronotropic medications such as beta-blockers for at least 48 hours before the exercise test to facilitate an appropriate (>90%) maximal heart rate on the day of assessment. Stress echocardiography was performed on a Lode B.V 917,900 (Angio Imaging with stress support) semi-recumbent bicycle with an incremental ramp protocol of 25 Watts every 2 minutes with simultaneous 12-lead ECG recording. Echocardiographic images were acquired at rest, 60% of maximal heart rate (220-age), 80% maximal heart rate and peak heart rate (defined as 90–100% of maximal predicted heart rate). Age-predicted maximal heart rate (220−age) was used to allow for ease of universal clinical application. Individuals were encouraged to maintain a constant cadence rate of 60–65 revolutions per minute during the first stage to achieve 60% of the maximal predicted heart rate. Subsequently, the cadence was increased to 75–80 revolutions per minute to reach 80% of maximal predicted heart rate. Thereafter, participants were requested to increase their cadence 85–90 revolutions per minute to ensure they achieved their peak heart rate. Blood pressure was manually recorded by auscultating the brachial artery using a mercury sphygmomanometer at rest and peak exercise.

At each stage, apical 4-chamber, apical 2-chamber, apical 3-chamber, parasternal long axis and parasternal short axis views at the level of the papillary muscles were taken in accordance with the recommendations provided by the British Society of Echocardiography [8]. Pulse wave Doppler and pulse tissue Doppler imaging were performed at the mitral valve inflow and mitral annulus, at rest, 60% of the maximal predicted heart rate and peak heart rate to assess diastolic filling pressures and relaxation. Right ventricular images were also obtained at rest and peak exercise in the apical 4-chamber view.

Live analysis was performed for regional wall motion abnormality at each stage. Offline calculations were performed to determine diastolic filling pattern, and the LV ejection fraction, which was calculated using the Simpson Biplane method from the apical 4-chamber and apical 2-chamber views at each stage in accordance with the British Society of Echocardiography standards [8]. Two independent physiologists measured LVEF on the same pre-selected high-quality cycles from the set of ≥ 5 consecutive cycles acquired. Cycles affected by respiratory translation or transient loss of the acoustic window due to lung artefact were excluded, as were any foreshortened beats. On average 3 cycles of each participant were excluded due to artefact.

### Strain analysis

Global longitudinal strain (GLS) analysis was conducted using images obtained during stress echocardiogram. At the time of image acquisition, the quality of the images was optimised for depth, gain and LV position.

GLS was assessed using the integrated GE platform at the time of image acquisition. Frame rates were optimised by obtaining frame rates between 60–70 at rest and increasing to > 80 frames/s at higher heart rates. Images of apical 4-chamber, apical 2-chamber, and apical 3-chamber views were analysed separately.

For each stage of exercise, only cardiac cycles with stable speckle tracking, continuous endocardial border delineation, and no segmental dropout were included. A minimum of 14/17 adequately tracked segments was required for GLS averaging [10–12].

Given the recognised limitations of speckle-tracking at very high heart rates (>140–150 bpm), peak-exercise GLS was interpreted descriptively and not used as a diagnostic endpoint.

### Statistical analysis

Statistical analysis was performed using SPSS (Version 29.0 and 30.0, IBM).

Data has been expressed as means ± standard deviation (SD) or percentages, where appropriate. Normality of continuous variables was assessed using the Shapiro-Wilk test. For continuous variables, group differences were analysed using either an independent samples t-test for normally distributed data or the Mann- Whitney U test for non-normally distributed data. Categorical variables were compared using the Fisher exact test or chi-squared test, as appropriate. For comparisons involving more than two groups, a one-way analysis of variance (ANOVA) was employed to identify differences across the three groups, followed by post-hoc tests where necessary. For pairwise comparisons between control athletes and athletic DCM individuals, unpaired T-tests were conducted to evaluate intra-group differences. Statistical significance was defined as a *p*-value < 0.05. Receiver operating characteristic (ROC) analysis was performed to evaluate the diagnostic performance of stress echocardiographic parameters, with area under the curve (AUC) and 95% confidence intervals calculated.

## Results

### Subject demographics

The mean age within the cohorts ranged from 32.5 to 37.3 years, with no significant differences amongst groups. Most participants were white (≥82%). There was a 3:1 male-to-female ratio amongst athletic DCM individuals and control athletes, and a 9:1 ratio in grey zone athletes. The cohort participated in a variety of sporting disciplines including, duathlon (19%), cycling (16.5%), running (14.8%), triathlon (12.6%), rowing (6%), and over 30% engaged in gym based or mixed sports (including rugby, basketball, football, hockey and gym based). Most grey-zone athletes participated in mixed sports, duathlon and triathlon (Table 1).

**Table 1:** Demographic profiles of athlete cohorts and their engagement in various sporting disciplines

	Controls Athletes	Athletic DCM individuals	Grey zone Athletes	p value
Number	62	58	62	
Age (mean years)	33.1 ± 12 (16–64)	35.9 ± 13 (16–65)	36.5 ± 13 (16–65)	0.3
Body surface area (m^2^)	1.95 ± 0.2 (1.5–2.4)	1.99 ± 0.2 (1.6–2.5)	2.00 ± 0.2 (1.4–2.4)	0.79
Hours of exercise per week	10.1 ± 4.8 (4–22)	8.44 ± 5.5 (4–30)	9.4 ± 5.4 (4–28)	0.22
White Ethnicity	57 (92%)	52 (89.7%)	52(83.9%)	0.61
Gym/Mixed sport	15 (24%)	24 (41.4%)	19 (30.6%)	0.11
Duathlon	7 (11%)	10 (17.2%)	18 (29%)	0.04
Cycling	14 (23%)	11 (18.9%)	7 (11.3%)	0.09
Running	7 (11%)	12 (20.8%)	8 (12.9%)	0.14
Triathlon	13 (21%)	1(1.7%)	10(16.2%)	<0.01
Rowing	6 (10%)	0	0	<0.01

Abbreviations: DCM = dilated cardiomyopathy

### 12-lead ECG

ECG abnormalities were more common in athletic DCM individuals (25.8%) than in controls (19.3%) and grey-zone athletes (17.7%; *p* < 0.001), with no significant difference between the latter two (*p* = 0.32). Twelve (19.3%) control athletes and 8 (12.9%) grey zone athletes revealed T wave inversion (TWI) in either the anterior (V2 and V3) or the inferior leads, or an isolated premature ventricular complex (PVC) with no evidence of evolving cardiomyopathy phenotype on imaging for over 5 years. Athletic DCM individuals with an abnormal ECG frequently revealed LBBB (13.8%), 5.2% showed lateral TWI, and 1 exhibited inferolateral TWI (Table 2). Neither the control nor the grey zone athletes exhibited non-sustained ventricular tachycardia compared with 8.1% athletes with DCM.

**Table 2:** ECG findings across athlete cohorts

	Control (N = 62)	DCM athletes (N = 58)	Grey-zone athletes (N = 62)	Control vs DCM athletes P-value	Control vs Grey zone athletes P-value	Across all Cohorts P-value
Sinus Rhythm	29 (46.8%)	39 (67.2%)	37 (59.7%)	0.06	0.11	0.09
Sinus Bradycardia	32 (51.6%)	18 (31.1%)	24 (38.7%)	0.57	0.11	0.07
Atrial Fibrillation	0 (0%)	1 (1.72%)	1 (1.60%)	0.46	1.00	0.57
Sinus tachycardia	1 (1.6%)	0 (0%)	(0) 0%	1.00	0.48	0.38
Mean HR	62.3	66.0	65.6	0.18	0.24	0.33
QRS duration (ms)	98.0	110.2	102.6	<0.01	0.03	0.01
Abnormal ECG	12 (19.3%)	18 (31%)	8 (12.9%)	<0.01	0.32	<0.01
LBBB	0 (0%)	8 (13.8%)	0 (0%)	<0.001	1.00	<0.01
Anterior or inferior T wave inversion	10 (16.1%)	6 (10.3%)	7 (11.3%)	0.17	0.81	0.29
PVCs on 12-lead ECG	2 (3.2%)	6 (10.3%)	1 (1.6%)	0.41	0.5	0.19

Abbreviations: DCM = dilated cardiomyopathy; ECG = electrocardiogram; HR = heart rate; LBBB = left bundle branch block; LVH = left ventricular hypertrophy; RBBB = right bundle branch block; TWI = T wave inversion; PVC = premature ventricular complexes

### Echocardiography

Resting LV dimensions and end-diastolic volumes were similar across groups, but grey-zone and athletic DCM individuals had higher end-systolic volumes than controls. Resting LVEF was higher in controls (61.1%) than in grey-zone (52.1%) and DCM individuals (53.1%; *p* = 0.005). Mitral annular systolic velocities were comparable, while septal and lateral E′ velocities were higher in controls and grey-zone athletes (Table 3). All participants achieved ≥ 90% of their age-predicted maximal heart rate. Mean peak heart rates did not differ significantly between groups (Controls: 178.9 ± 13.0 bpm [95.4 ± 2.9% of predicted]; DCM: 175.4 ± 12.8 bpm [95.7 ± 3.4%]; Grey Zone: 177.9 ± 11.0 bpm [96.9 ± 3.8%]; *p* = 0.291), confirming comparable exercise intensity across groups.

**Table 3:** Resting echocardiographic and CMR parameters in the athlete cohorts

	Control athletes (N = 62)	Athletic DCM Individuals (N = 58)	Grey Zone athletes (N = 62)	P Control vs DCM	P Control vs Grey-zone	P All cohorts
**ECG**						
Abnormal ECG - International criteria	19.3%	31%	12.9%	<0.01	0.32	<0.01
**Ambulatory Monitor**						
Ambulatory - NSVT	0%	8.1%	0%	<0.01	1.00	1.39
**ECHO**						
LV end-diastolic Diameter (mm)	57.6 ± 1.2 (52–64)	57.6 ± 1.3 (56–70)	58.2 ± 1.2 (52–66)	0.14	0.01	0.43
LV end-diastolic volume (ml)	168.5 ± 39 (124–310)	160.2 ± 45 (121–289)	176.1 ± 32 (122–242)	0.35	0.3	0.16
LV end-diastolic indexed volume (ml/m^2^)	84.1 ± 17 (71–127)	80.7 ± 19 (71–152)	85.4 ± 15 (71–122)	0.38	0.67	0.41
LV end-systolic volume (ml)	63.9 ± 17 (48–77)	77.8 ± 27 (48–167)	75.4 ± 17 (48–120)	<0.01	<0.01	<0.01
LV end-systolic indexed volume (ml/m^2^)	32.6 ± 6 (28–48)	38.3 ± 11 (28–66)	36.2 ± 8 (28–60)	<0.01	0.03	0.01
Resting LVEF Biplane (%)	61.1 ± 4 (55–73)	53.1 ± 5 (45–55)	52.1 ± 3 (45–54)	<0.01	<0.01	<0.01
LA diameter (mm)	38.1 ± 5 (28–51)	36.9 ± 5 (23–50)	37.6 ± 5 (22–54)	0.33	0.67	0.64
LA volume Biplane (ml)	83.6 ± 23 (36–139)	73.9 ± 30 (29–158)	79.8 ± 21 (36–133)	0.10	0.47	0.23
Resting lateral e’ (m/s)	0.18 ± 0.04 (0.07–0.23)	0.10 ± 0.05 (0.03–0.22)	0.15 ± 0.03 (0.08–0.22)	0.01	0.67	0.02
Resting lateral S (m/s)	0.11 ± 0.03 (0.06–0.2)	0.07 ± 0.2 (0.05–0.12)	0.10 ± 0.03 (0.03–0.2)	0.10	0.46	0.23
Resting septal e’ (m/s)	13.1 ± 3 (6–18)	9.7 ± 4 (2–19)	11.3 ± 3 (7–17)	0.01	0.03	0.01
Resting septal S (m/s)	0.09 ± 0.02 (0.05–0.12)	0.08 ± 0.03 (0.04–0.17)	0.09 ± 0.01 (0.06–0.12)	0.12	0.95	0.13
Resting E (m/s)	0.07 ± 0.01 (0.05–0.11)	0.07 ± 0.02 (0.03–0.10)	0.06 ± 0.02 (0.04 -0.09)	0.09	0.02	0.07
Resting A (m/s)	0.05 ± 0.01 (0.03–0.07)	0.05 ± 0.02 (0.02–0.09)	0.05 ± 0.01 (0.03–0.07)	0.03	0.72	0.02
Resting E/A ratio	1.7 ± 0.4 (0.8–2.4)	1.3 ± 0.6 (0.4–2.7)	1.4 ± 0.4 (0.8–2.5)	0.01	0.01	0.02
Deceleration Time (ms)	243.9 ± 76 (20–368)	258.6 ± 85 (123–572)	233.8 ± 63 (128–355)	0.49	0.56	0.41
**CMR**						
Indexed LV volume (ml/m^2^)	109 ± 12 (101–164)	113 ± 10 (103–317)	112 ± 10 (102–149)	0.31	0.23	0.7
LVEF (%)	63.7 ± 4 (55–73)	54.66 ± 5 (44–66)	54.6 ± 2.4 (46–57)	<0.001	<0.001	<0.01
Major scar (%)	4.8%	34.4%	9.6%	<0.001	<0.001	<0.01
**CPET**						
Mean VO2 max (ml/min/kg)	48.1 ± 8.1 (32.5–68.7)	33.5 ± 7.2 (20.1–48.9)	42.4 ± 8.9 (24–65.7)	<0.01	<0.01	<0.01

Abbreviations: DCM = dilated cardiomyopathy; CMR = cardiovascular magnetic resonance; CPET = cardiopulmonary exercise test; ECHO = echocardiogram; LA = left atrium; LV = left ventricle; LVEF = left ventricular ejection fraction; NSVT = non-sustained ventricular tachycardia

### CMR

There were no significant differences in the mean indexed LV volumes between control athletes and DCM individuals. Similar to the echocardiogram, LVEF was higher amongst controls (63.7%) than grey zone (54.6%) and DCM individuals (54.6%); *p*=<0.001). Major fibrosis was observed in 3 (4.8%) control athletes, 20 (34.4%) DCM individuals and 6 (9.6%) grey zone athletes (Table 3).

### Stress echocardiography

#### Change in LVEF from rest to peak exercise (ΔLVEF)

**Fig. 1:**
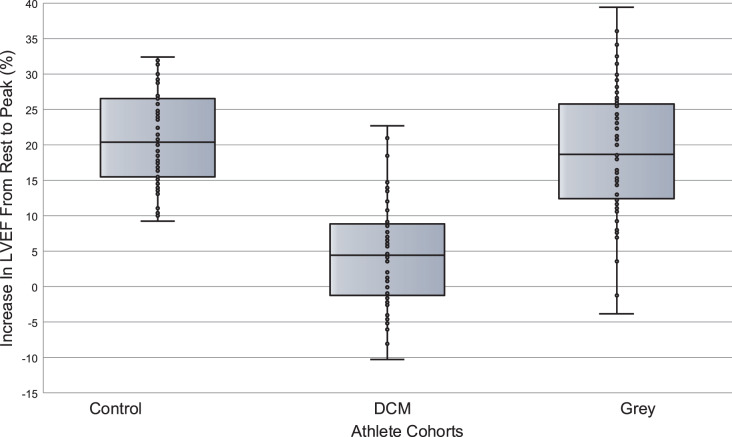
Box plot demonstrating the distribution of LVEF augmentation from rest to peak in the control, athletes with DCM and grey zone athlete cohorts. Abbreviations: DCM = Dilated cardiomyopathy; LVEF = left ventricular ejection fraction

**Fig. 2:**
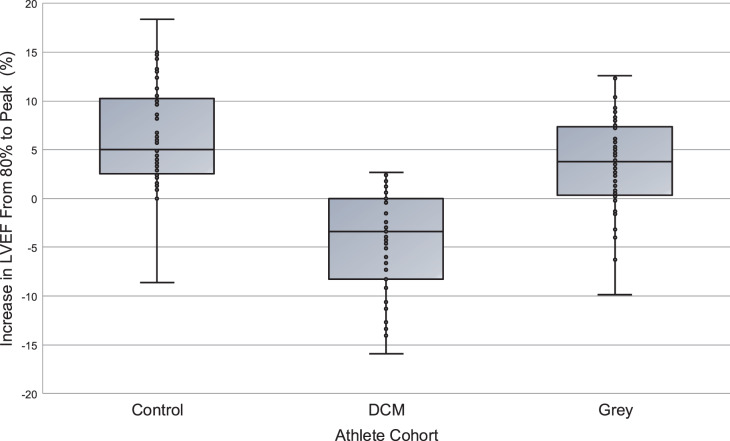
Boxplot distribution of LVEF augmentation from submaximal to maximal exercise. Abbreviations: DCM = Dilated cardiomyopathy; LVEF = left ventricular ejection fraction

Augmentation of LVEF from rest to peak exercise was significantly greater in controls (Δ LVEF 21%) and grey-zone athletes (Δ LVEF 19.2%) than in athletic DCM individuals (Δ LVEF 4.9%; *p* < 0.001), with control athletes showing slightly more significant augmentation than grey-zone athletes (*p* < 0.001) Table 4; Fig. 1. The mean augmentation from submaximal (up to 80% of the age-predicted heart rate) to maximal exercise was 7.5% in control athletes and 3.5% in grey zone athletes (*p* < 0.001). In contrast, athletic DCM individuals showed a mean decline in LVEF of −4.3% (*p* < 0.001) (Table 4, Fig. 2).

**Table 4:** Stress echocardiography indices in control, DCM and grey zone athlete

	Control Athletes	Athletic DCM Individuals	Grey zone Athletes	P Control vs DCM	P Control vs Grey P	P All cohorts
Peak Heart Rate Achieved	178.9 ± 13.0 (150–207)	175.4 ± 12.8 (148–203)	177.9 ± 11.0 (154–200)	0.11	0.8	0.25
% of Maximal Heart Rate Achieved	95.4 ± 2.9 (91–102)	95.7 ± 3.4 (91–106)	96.9 ± 3.8 (91–108)	0.13	0.43	0.21
Resting LVEF Biplane (%)	62.1 ± 4.6 (55–73)	53.1 ± 5.7 (45–55)	52.1 ± 3 (45–54)	<0.01	0.01	<0.01
60% max HR LVEF Biplane (%)	68.7 ± 6.1 (51–81)	58.1 ± 9.1 (31–76)	61.5 ± 7.7 (35–76)	<0.01	0.04	<0.01
80% max HR LVEF Biplane (%)	77.1 ± 5.1 (64–87)	62.2 ± 5.1 (42–84)	71.7 ± 7.4 (54–87)	<0.01	<0.01	<0.01
Peak LVEF Biplane (%)	83.1 ± 4.9 (73–92)	57.8 ± 11.2 (33–83)	74.5 ± 9.5 (50–91)	<0.01	<0.01	<0.01
Δ LVEF Rest to peak (%)	21 ± 6.6 (9.3–32)	4.9 ± 8.1 (−10–30)	19.2 ± 8.8 (−3.8–39)	<0.01	<0.01	<0.01
Δ LVEF submaximal to peak (80% max HR to peak HR (%)	7.5 ± 11.9 (−8–18)	−4.3 ±6.3 (−22–17)	3.3 ± 6.3 (−17–26)	<0.01	<0.01	<0.01
Mean GLS at rest (%)	−18.9 ±2.1 (−24.7- −13.8)	−15.3 ±2.3 (−20.5- −9.9)	−17.5 ±2.5 (−22.2- −13.3)	<0.01	<0.01	<0.01
Mean GLS at peak (%)	−16.9 ±2.9 (−21.6- −9.3)	−12.9 ±2.8 (−12.9- −18)	−15.9 ±2.7 (−15.8 - −21.2)	<0.01	0.15	<0.01
Δ GLS Rest to peak (%)	2.3 ± 3.6 (−4.6- 17.5)	3.1 ± 4.3 (−6.10- −16)	3.1 ± 5.3 (−4.4- 16.9)	0.31	0.351	0.65

Abbreviations: DCM = Dilated cardiomyopathy; GLS = global longitudinal strain; LV = left ventricle; Max = maximum

Interobserver variability for ΔLVEF was low. Using these measurements, the standard deviation of ΔLVEF was 0.8%, with coefficients of variation of 1.31% (controls), 1.51% (DCM), and 1.44% (grey zone). Accounting for this degree of interobserver variation, ongoing augmentation from 80% predicted maximal heart rate to peak exercise remained a reproducible discriminator of athletic DCM.

**Fig. 3:**
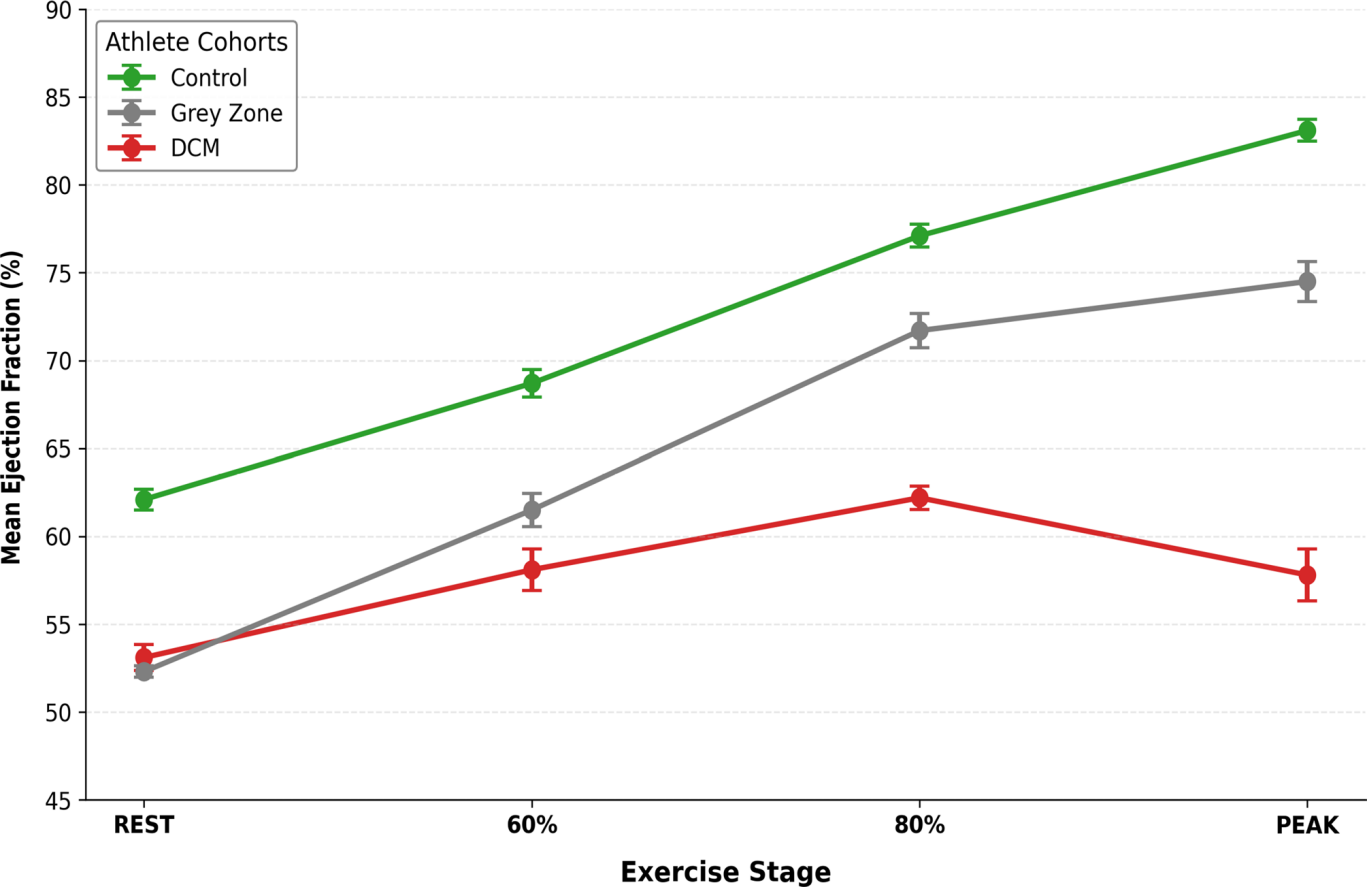
Trend of LV augmentation at each exercise stage for the different athlete cohorts. Abbreviations: DCM = dilated cardiomyopathy

Most (98.3%) control and grey-zone athletes (90.3%) met both LVEF augmentation thresholds (≥10% from rest to peak and an ongoing increase from submaximal to peak exercise). In comparison, only 20.6% of DCM individuals augmented ≥ 10% from rest to peak, and 98% showed a drop from submaximal to peak exercise (Fig. 3).

Importantly, 13 out of 17 (76%) of those with DCM with restored LV systolic function following pharmacotherapy failed to augment from rest to peak and all 17 individuals were unable to augment their LVEF from submaximal to peak.

Utilising the inability to augment > 11% threshold from our pilot study [5] identified DCM with a sensitivity of 86.5% and a specificity of 90.3%. In comparison, applying the ≥10% threshold recommended by the ESC sports cardiology guidelines [6]. yielded a lower sensitivity (79.4%) but significantly higher specificity (98.3%) for identifying DCM. Combining the inability to augment LVEF by ≥10% from rest to maximal exercise with the failure to augment from submaximal to maximal exercise identified DCM with the highest diagnostic accuracy, yielding a sensitivity of 98.1% and a specificity of 98.4%. ROC analysis confirmed superior discriminatory performance for the combined criterion (AUC 0.942, 95% CI 0.890–0.994) compared with the ≥10% threshold alone (AUC 0.888, 95% CI 0.815–0.960) and ΔLVEF as a continuous variable (AUC 0.926, 95% CI 0.871–0.981; all *p* < 0.001). Logistic regression confirmed both ΔLVEF and submaximal-to-peak augmentation as independent predictors of DCM (ΔLVEF: OR 0.795, *p* < 0.001; submaximal-to-peak: OR 0.765, *p* = 0.004; Nagelkerke R^2^ 0.775).

### Sex differences

This study comprised of 33 females, including 14 control athletes, 13 athletic DCM individuals and 6 grey-zone athletes. There were no significant sex differences in ΔLVEF among control (males: 21.1% vs. females: 20.7%, *p* = 0.8) or grey zone athletes (males: 19.3% vs. females: 19.5%, *p* = 0.9). However, among athletic DCM individuals, males demonstrated a higher mean ΔLVEF compared to their female counterparts (6.2% vs. 1.1%, *p* = 0.05).

### Exercise responses in relation to myocardial fibrosis

Among the grey-zone athletes, 6 (9.6%) revealed major fibrosis, exclusively seen in the midwall of the inferolateral or subepicardial wall on CMR. There were no differences in exercise responses between grey-zone athletes with fibrosis compared to those without fibrosis. Five of the 6 (83.3%) grey zone athletes with fibrosis exhibited a ΔLVEF ≥ 10% from rest to peak exercise and the ability to augment from submaximal effort to peak heart rate compared with 54 (90%) grey zone athletes without fibrosis (*p* = 0.43).

### Strain evaluation

Control and grey zone athletes demonstrated a significantly more negative resting and peak mean GLS than DCM individuals (−18.9% and −17.5% vs. −15.3%; *p* < 0.001) and (−16.9% and −15.9%, vs 12.9%; *p* < 0.001) respectively. There were no significant differences in the mean change of average GLS from rest to peak among the healthy controls, grey zone athletes and DCM individuals (+2.3%, +3.1%, and +3.1%, respectively; *p* = 0.635).This uniform reduction likely reflects recognised limitation of speckle-tracking at high heart rates rather than a physiological decline in contractility. Using an abnormal GLS cutoff of ≤−17.5% (upper quartile threshold in controls), 9.6% of controls, 34% of grey-zone athletes, and 88.4% of DCM individuals had abnormal values

## Discussion

An enlarged LV and mildly depressed LVEF, which necessitates the differentiation between the athlete’s heart and DCM, is one of the most common clinical dilemmas in sports cardiology. A study of 286 elite male professional cyclists participating in Tour de France competitions reported an LV cavity size ≥ 60 mm in 50%. Over 80% of cyclists with an LV cavity ≥ 60 mm had an LVEF ≤ 52% [2]. A more recent CMR study of 281 endurance cyclists and runners showed that 11% of athletes had a LVEF < 50% [7]. The authors reported excellent myocardial reserve in their grey zone athletes, with a mean ΔLVEF of 18%, however, exercise CMR is not readily available. In such cases, demonstrating good myocardial reserve on exercise echocardiography may be a more pragmatic method of differentiating physiological adaptation from DCM. The precise echocardiographic threshold for ΔLVEF in distinguishing physiological LV enlargement from DCM in athletes with a dilated LV and mildly depressed LVEF remains ambiguous. This uncertainty arises from the paucity of studies and the limited number of study participants, as few DCM individuals can engage in vigorous exercise or participate in dynamic competitive sports.

Our prior study comparing 35 asymptomatic athletic DCM individuals and 25 male grey zone athletes revealed that an increase in LVEF > 11% during exercise differentiated physiological LV enlargement from DCM with a sensitivity and specificity of 77.1% and 96%, respectively [5].

In this larger multicentre study, we revealed that a large proportion of grey zone athletes (90.3%) demonstrated the ability to augment their LVEF by ≥10%, compared to only 20.6% of DCM individuals. We examined the additional value of augmenting LVEF between 80% maximal HR and peak heart rate, on the understanding that an increasing preload in DCM may reduce the efficiency of myocardial contraction, compatible with Starling’s theory [13–15]. We showed that most (98%) DCM individuals typically revealed a decline in LVEF from submaximal to maximal exertion, even among those who achieved ≥ 10% increase in LVEF. Combining the inability to increase LVEF ≥ 10% or an increase in LVEF from submaximal to peak exercise was associated with a higher sensitivity and specificity for diagnosing DCM. Sex did not influence LVEF augmentation during exercise. Our study utilised a technique requiring semi-supine bicycle imaging, which may limit its applicability in centres using treadmill-based protocols.

A subset of grey zone athletes (9.6%) failed to demonstrate the expected augmentation response (Δ LVEF ≥ 10%), and 6 had myocardial scarring. However, there were no differences in the ability to augment between the small group with major fibrosis and those without, suggesting that localised fibrosis may not necessarily impair contractile reserve in this population.

In our previous study, we emphasised that an inability to achieve a peak LVEF ≥ 63% as a marker of DCM. In this study, all healthy athletes achieved a peak LVEF ≥ 72%, however, this response was observed in only 63.2% of grey zone athletes and also in 11.5% of athletic DCM individuals. Given the lower resting LVEF observed in grey zone athletes, achieving a peak LVEF ≥ 72% would require a substantially greater contractile reserve than control athletes. Therefore, we recommend placing more focus on Δ LVEF than peak LVEF.

Resting 2D-global longitudinal strain (GLS) has been proposed as a potential discriminator between physiological LV remodelling and DCM. However, collective experience indicates that GLS has limited utility in clarifying contractile function in healthy athletes with a reduced LVEF. Our prior study demonstrated that 14 (58.3%) control athletes, 27 (79.4%) DCM individuals, and 17 (68%) of grey zone athletes exhibited low GLS values (defined as >−17.5%) [5]. The current study found 6 (9.6%) control athletes, 46 (88.4%) DCM individuals and 23 (34%) of grey zone athletes had low GLS (>−17.5%). Crucially, the change in GLS from rest to peak exercise did not differ between groups, indicating that ΔGLS lacks discriminatory value. This behaviour likely reflects the reduced reliability of speckle tracking at higher heart rates, rather than true physiological differences. Collectively suggesting that average resting GLS would lead to a high number of false positive results at rest, and ΔGLS would have no discriminative value following exercise.

## Limitations

This study presents several limitations that deserve attention. While our investigation of grey zone athletes with a dilated left ventricle and mildly impaired resting LVEF included more participants than previous studies, the overall sample size remains relatively modest. Consequently, larger multicentre studies are necessary to further explore this issue. Our research primarily involved predominantly male and white individuals, which may limit the applicability of the findings to other ethnic groups.

We included a small number of grey zone athletes with myocardial fibrosis (*n* = 8), which may indicate DCM. However, emerging evidence suggests that approximately 13% of lifelong male athletes display subepicardial or mid-wall scarring, typically affecting the basal inferolateral wall, without significant implications [9, 16]. Furthermore, all but one grey zone athlete with myocardial scar showed good myocardial reserve. A proportion of grey zone athletes were considered to have ECG anomalies warranting further investigation. The prevalence and qualitative differences in the anomalies were similar to those of the control group. Anterior T-wave inversion confined to V3 has been reported in healthy athletes in several studies [17, 18], and there is emerging data that isolated T wave inversion in the inferior leads is relatively nonspecific with low diagnostic yield [19]. Given the current understanding, it would be premature to classify these individuals as having DCM until further data is available. Some grey-zone athletes did not meet our criteria for adequate myocardial reserve, suggesting they may progress to DCM in the future. This cross-sectional study requires further longitudinal evaluations for this subgroup and prospective follow-up of this cohort is currently ongoing, which may provide further insight into the prognostic utility of stress echocardiography and help clarify the natural history of contractile reserve impairment and its relationship to the development of cardiomyopathy in this population.

Our grey zone cohort included only 6 females, which is not unexpected since the diagnostic challenges of an enlarged LV with a depressed resting LVEF primarily concern male athletes. Nevertheless, dedicated studies focusing on female athletes are necessary to address this knowledge gap. We did not conduct genetic testing on our grey zone athletes, which may have been of value in a small proportion of cases.

Sport type differed numerically between groups, with DCM individuals more commonly engaged in gym-based or mixed sports, though these differences did not reach statistical significance.

The low interobserver variability reported in this study reflects offline measurements by experienced cardiac echocardiographers within a standardised department, on pre-selected high-quality loops. This level of reproducibility may be more challenging to achieve in routine clinical settings with less experienced operators and without established echocardiogram reporting standardisation.

## Conclusion

**Fig. 4:**
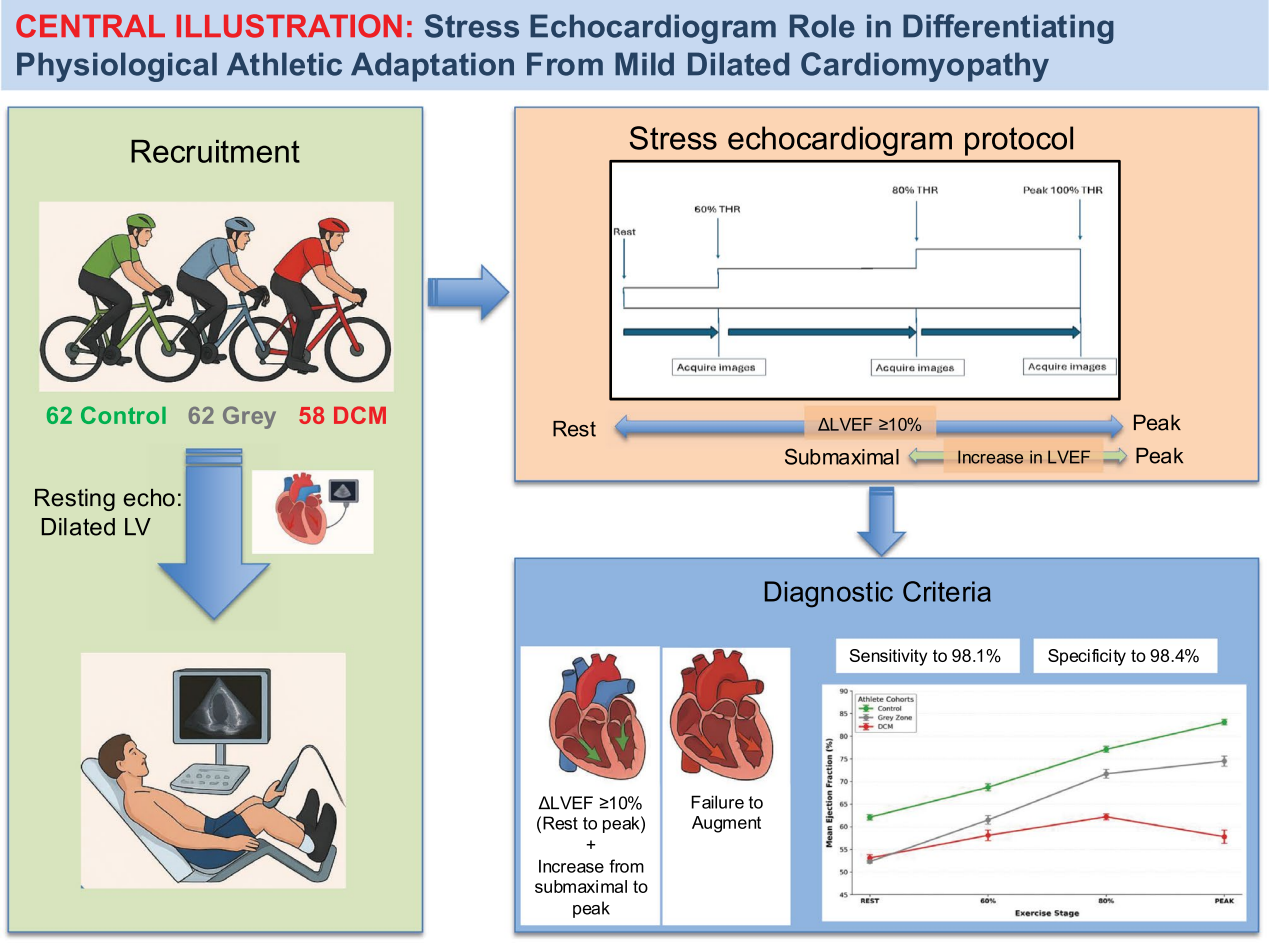
Central illustration: summary of study protocol and improved sensitivity and specificity when using submaximal and peak exercise testing. Abbreviations: DCM = dilated cardiomyopathy; LV = left ventricle; LVEF = left ventricular ejection fraction

Stress echocardiography is a pragmatic second-line investigation for distinguishing between physiological LV enlargement and dilated cardiomyopathy (DCM) in athletes presenting with the grey zone phenotype. A failure to achieve an increase in LVEF ≥ 10% from rest to peak exercise, including increase in LVEF from submaximal to maximal effort, differentiates DCM from physiological LV remodelling with high sensitivity and specificity (Fig. 4).

## Data Availability

The datasets generated and analysed during the current study contain clinical information and are therefore not publicly available. Data can be made available from the corresponding author on reasonable request, subject to institutional governance and reasearch and ethical authorisation and data-sharing agreements, and only in a de-identified form. No patient-level data can be shared publicly due to privacy and ethical restrictions
